# Antiretroviral treatment outcomes from a nurse-driven, community-supported HIV/AIDS treatment programme in rural Lesotho: observational cohort assessment at two years

**DOI:** 10.1186/1758-2652-12-23

**Published:** 2009-10-08

**Authors:** Rachel Cohen, Sharonann Lynch, Helen Bygrave, Evi Eggers, Natalie Vlahakis, Katherine Hilderbrand, Louise Knight, Prinitha Pillay, Peter Saranchuk, Eric Goemaere, Lipontso Makakole, Nathan Ford

**Affiliations:** 1 Médecins Sans Frontières, Morija, Lesotho; 2 Médecins Sans Frontières, Cape Town, South Africa; 3 Infectious Diseases Epidemiology Unit, University of Cape Town, South Africa; 4 Scott Hospital, Morija, Lesotho

## Abstract

**Introduction:**

Lesotho has the third highest HIV prevalence in the world (an adult prevalence of 23.2%). Despite a lack of resources for health, the country has implemented state-of-the-art antiretroviral treatment guidelines, including early initiation of treatment (<350 cells/mm^3^), tenofovir in first line, and nurse-initiated and managed HIV care, including antiretroviral therapy (ART), at primary health care level.

**Programme approach:**

We describe two-year outcomes of a decentralized HIV/AIDS care programme run by Doctors Without Borders/*Médecins Sans Frontières*, the Ministry of Health and Social Welfare, and the Christian Health Association of Lesotho in Scott catchment area, a rural health zone covering 14 clinics and one district hospital. Outcome data are described through a retrospective cohort analysis of adults and children initiated on ART between 2006 and 2008.

**Discussion and Evaluation:**

Overall, 13,243 people have been enrolled in HIV care (5% children), and 5376 initiated on ART (6.5% children), 80% at primary care level. Between 2006 and 2008, annual enrolment more than doubled for adults and children, with no major external increase in human resources. The proportion of adults arriving sick (CD4 <50 cells/mm^3^) decreased from 22.2% in 2006 to 11.9% in 2008. Twelve-month outcomes are satisfactory in terms of mortality (11% for adults; 9% for children) and loss to follow up (8.8%). At 12 months, 80% of adults and 89% of children were alive and in care, meaning they were still taking their treatment; at 24 months, 77% of adults remained in care.

**Conclusion:**

Despite major resource constraints, Lesotho is comparing favourably with its better resourced neighbour, using the latest international ART recommendations. The successful two-year outcomes are further evidence that HIV/AIDS care and treatment can be provided effectively at the primary care level. The programme highlights how improving HIV care strengthened the primary health care system, and validates several critical areas for task shifting that are being considered by other countries in the region, including nurse-driven ART for adults and children, and lay counsellor-supported testing and counselling, adherence and case management.

## Introduction

Lesotho has the third highest HIV prevalence in the world (after Swaziland and Botswana), at 23.2% among adults aged 15 to 49 [1], and is the poorest of the three countries, ranking 138 out of 177 nations on the Human Development Index [2]. More than half of Lesotho's 1.8 million inhabitants live below the poverty line [2]. According to the latest available census data, Lesotho's population is declining drastically, largely as a result of HIV/AIDS [3,4].

The high HIV prevalence can be explained largely by Lesotho's dependence on migrant labour: the heavy reliance on remittances from miners employed in South Africa, by some estimates, accounts for almost 60% of Lesotho's gross domestic product [5]. One in every three male wage earners works in South Africa. This dependence on migrant labour - characterised by unsafe and unhealthy working conditions [6], overcrowded living quarters, long periods away from family and community, and easy access to commercial sex work [7,8] - is driving the dual epidemics of HIV and tuberculosis (TB).

An estimated 270,000 people are living with HIV/AIDS in the country, and between 80,000 and 85,000 of these are estimated to be in clinical need of antiretroviral therapy (ART) [9]. HIV/AIDS is having a devastating impact on all aspects of Basotho society, including health, education, agriculture and general economic development. It is the leading cause of mortality, accounting for 56% of deaths among children under five [10], and is responsible for a more than 20 year drop in life expectancy over the past two decades - to as low as 36 years, according to recent statistics [11]. Approximately 18,000 people die annually of AIDS-related complications, representing 1% of the entire population [12].

In addition to its HIV/AIDS epidemic, Lesotho also has the fourth highest TB incidence in the world (635 per 100,000 people per year [13]); according to estimates from the Lesotho National Tuberculosis Programme, up to 90% of TB patients are also infected with HIV. The high co-infection rate, the historically weak TB programme, and the presence of multidrug and extensively drug resistant (M/XDR) TB in every province in neighbouring South Africa has created conditions for a dire drug-resistant (DR) TB problem in Lesotho: 10% of patients with smear-positive TB in Lesotho are estimated to have multidrug-resistant TB [14].

The Government of Lesotho has shown strong commitment to addressing HIV/AIDS and TB. However, health care delivery has been severely limited by major resource constraints, in particular a dire shortage of professional health workers: there are just five doctors and 62 nurses per 100,000 inhabitants in Lesotho (neighbouring South Africa has 74 doctors and 393 nurses per 100,000 inhabitants) [15]; 80% of doctors in Lesotho are visiting foreigners, mainly from other parts of Africa, awaiting certification to practice in South Africa; around a quarter of nurses leave their posts to seek work elsewhere; and another quarter of nurse attrition is due to death.

In January 2006, *Médecins Sans Frontières *(MSF) and the Ministry of Health and Social Welfare (MOHSW) launched a joint pilot programme to provide decentralized HIV/AIDS care and treatment at the primary health care level. The programme, which relies on a nurse-driven approach, was launched in what was formerly called Scott Hospital Health Service Area, a rural health zone straddling Maseru and Mafeteng districts, with a population in the catchment area of approximately 200,000 people.

This article describes the development, evolution and main outcomes of the first three years of this programme.

## Programme approach

The decentralized model of care developed in Scott catchment area covers one 102-bed district hospital and 14 basic, rural health centres, each staffed only by nurses. These nurses are responsible for providing all primary health care and for integrating a full range of HIV/AIDS services, including HIV testing and counselling (HTC), prevention of mother to child transmission (PMTCT) services, TB and HIV care, and antiretroviral therapy, into the package of primary health care offered at the health centre level.

At the start of the programme, approximately 30,000 people were estimated to be living with HIV/AIDS in Scott catchment area. Knowledge of clinical management of HIV was limited and few drugs to treat opportunistic infections were available; ART was not available at all. Building on MSF's previous experience in South Africa [16], nurses were supported to initiate and manage HIV care and ART at the health centres. Unlike South Africa, the Lesotho health authorities encouraged task shifting to enable all levels of nurses with diagnosing, prescribing and dispensing powers; this model was readily accepted by the MOHSW for replication throughout the country (Table 1).

**Table 1:** Allocation of HIV and TB tasks for doctors, nurses and lay counsellors at primary health care level

**District level**	**Tasks**
Public health nurse	- Carries out monthly visits to health centres - Conducts quarterly supervision visits - Provides refresher trainings
Doctor	- Provides clinical mentorship at health centres (and OPD) during bi-weekly clinic visits - Provides referral support for complicated cases - Prescribes ART for non-ARV naïve patients - Prescribes TB treatment for HIV+ patients with sputum negative and/or EP TB in a patient who is in the first three months of ART - Manages patients suspected to have TB IRIS - Makes clinical decision about switching to second-line therapy, as needed - Manages Grade 4 side effects - Formally admits patients to hospital and provides inpatient care
**Health centre level** **Nurse clinician** MOHSW minimum staffing: 1 per health centre	- Initiates and manages first-line ART for adults and children - Interprets chest x-rays to diagnose smear negative TB using the smear negative algorithm and detects unilateral pleural effusion and miliary patterns (if specifically trained) - Initiates second-line ART in the case of treatment failure, after doctor's approval - Interrupts treatment in the case of severe adverse events and manages treatment substitutions for first line as needed - All of the below
**Professional nurse** MOHSW minimum staffing: 1 per health centre	- Initiates and manages first-line ART for adults and children - Makes a presumptive diagnosis of severe HIV disease in children <18 months (in the absence of DNA PCR) - Refers patients to hospital - Initiates isoniazid prophylaxis - Initiates TB treatment for patients newly initiated on ART - All of the below
**Trained nurse assistant** MOHSW minimum staffing: 2 per health centre	- Initiates and manages first-line ART for adults and children - Stages HIV+ adults and children according to WHO classification and determines clinical need for ART - Manages opportunistic infections - Initiates cotrimoxazole as prophylaxis - Initiates short-course AZT prophylaxis for PMTCT - Prepares children's caregivers to provide ART - Provides education and counselling on feeding options for HIV+ pregnant women - Identifies DR-TB suspects and orders DST
**HIV/TB lay counsellor (adherence)** Recommended minimum staffing: 1 per health centre	- Provides preparatory counselling before patients are initiated on ART - Provides ART and TB treatment adherence counselling - Identifies TB and ART defaulters and mobilises community-based health workers to trace them - Facilitates support groups and provides health talks on pertinent topics (e.g., ANC and PMTCT, HTC, TB, ART) - Counsels pregnant women on PMTCT and testing schedule for infants - Schedules appointments for HIV patients, including: labs, counselling, refills and clinical exams, according to national guidelines - Assists in recording basic information in registers and compiling monthly reports, including pre-ART, ART, HTC, PMTCT, TB suspect, and general TB registers - Manages folders of HIV patients and files/cards of TB patients - All of the below
**HIV/TB lay counsellor (HTC)** Recommended minimum staffing: 1 per health centre	- Provides HIV testing and counselling for adults and children via rapid tests - Collects dried blood spots for PCR testing of infants, after training - Provides TB and STI screening and refers to nurse accordingly for all HIV+ patients - Weighs patients, carries out basic cough triage and other clinic support tasks - Provides prevention education and commodities - Provides sputum production education, fills out lab specimen request forms, collects and prepares lab samples for transport
**Community-based health worker**	- Traces TB treatment and ART defaulters - Provides education and encourages uptake of HIV- and TB-related services - Refers symptomatic patients to health centre - Carries out awareness-raising activities

DST: Drug sensitivity testing

IRIS: Immune reconstitution inflammatory syndrome

OPD: Outpatient department

PCR: Polymerase chain reaction

STI: Sexually transmitted infection

To equip nurses with the skills to meet these new responsibilities, intensive in-service theoretical and practical training was provided on management of HIV-related conditions and ART. This included quarterly "out-of-service" trainings, each lasting one week, which were clinical trainings adapted from the World Health Organization's (WHO's) Integrated Management of Adolescent and Adult Illness (IMAI) [17].

Targeted trainings were also provided on the basis of weaknesses identified via pre- and post-test evaluations and in-service support and supervision visits. These covered specific issues, such as drug management, monitoring and evaluation, laboratory investigations, diagnosis of smear-negative TB, DR-TB, infection control, family planning, isoniazid prophylaxis, PMTCT, and paediatric ART. In addition, a number of clinical support tools were developed, including a nurse-oriented guideline for HIV management [18], an algorithm for the diagnosis of smear-negative TB [19], and standardised protocols and flowcharts for basic clinic procedures.

Each clinic is staffed by just one or two nurses (often, they are nursing assistants with just two years of training), who provide a full range of primary care activities. Their work is supported by a doctor or an experienced nurse clinician, who visits on a weekly or bi-weekly basis to provide clinical mentorship for nurses on such issues as: the diagnosis and management of complicated HIV-related conditions, antiretroviral (ARV) side effects, and other clinical challenges; referral support for complicated cases; and assistance with general clinic management, including monitoring and evaluation tasks.

Nurse workload is high. An assessment in August 2006 found that nurses were carrying out up to 45 consultations per day, far greater than the WHO recommended maximum of 30 consultations per day (excluding HIV consultations). Acknowledging that the ever-increasing need for ART could not be met due to scarcity of doctors, nurses and other professional health staff, MSF and Scott Hospital established a cadre of HIV/TB lay counsellors to reinforce capacity to deliver HIV and TB services.

In contrast to traditional models of community-based health worker support, these lay counsellors (typically people living openly with HIV/AIDS) are facility based, receive structured training in HIV and TB and counselling, have clear task descriptions, and are compensated for their work, receiving 39 to 55 maloti (US$5-7) per day. As of July 2009, there were a total of 42 facility-based lay counsellors working across the catchment area.

Lay counsellors manage HTC services and provide pre-ART preparatory counselling, and ART and TB treatment adherence support. They also carry out general clinic support tasks, including tracking of patients who are eligible for ART but have not yet been started, and organising ART and TB defaulter tracing. One of the challenges they face, and an important barrier to adherence, is that many Basotho move temporarily or semi-permanently to South Africa in search of work.

Clinic staffers, including counsellors, try to respond to their clients' needs by detailing HIV clinical history in patient-held records, providing two to three month refills, and helping the client's continuity of care by discussing what facilities provide ART care in the area in South Africa to which they are moving.

## Discussion and Evaluation

### ART outcomes

ART was introduced at the primary care level in Scott catchment area in March 2006. As of July 2009, 13,243 people had been enrolled in HIV care (5% children), and 5376 initiated on ART (6.5% children), 80% at primary care level. Overall, four in five people are initiated at the health centre level. Enrolment has increased substantially year to year, while the proportion of adults arriving sick (with a CD4 count of less than 50 cells/mm^3^) has halved, from 22.2% in 2006 to 11.9% in 2008, an indication that people are seeking treatment earlier.

Outcomes for the first two years are satisfactory, with 80% of patients still alive and in care (i.e., still receiving ART) at 12 months, and 76.5% of patients remaining in care at 24 months (Table 2). These data compare favourably with outcomes from other programmes in Africa: a systematic review of HIV cohorts from 13 countries in sub-Saharan Africa reported lower retention rates at six months (79% vs 89% in Scott catchment area), 12 months (75% vs 80%) and 24 months (61.6% vs 77%) [20].

**Table 2:** Cumulative ART outcomes (adults and children)

	Adults	Children
Total enrolled	4061	244

12 months		

Cum lost-to-follow up	185 (8.8%)	2 (1.6%)

Cum died	234 (11.1%)	11 (8.9%)

Cum transferred	63	5

**Cum remaining in care at 12 months**	**1691 (80.1%)**	**110 (89.4%)**

24 months		

Cum lost-to-follow up	76 (9.4%)	1 (2.1%)

Cum died	114 (14.1%)	5 (10.4%)

Cum transferred	32	6

**Cum remaining in care at 24 months**	**618 (76.5%)**	**40 (87.5%)**

Although long-term follow-up data are still limited, the fact that three in four patients are still in care at two years is particularly encouraging and likely reflects the positive impact of two main programme principles, both of which have been associated with better retention in care: the decentralization of services to provide care close to people's homes, and the provision of free care [21-23].

Outcomes in children are also highly satisfactory, with 89.4% remaining in care at 12 months and 87.5% at 24 months. These data compare favourably with other programmes in southern Africa (Rwanda: 95% [24]; Malawi: 72% [25]) and data from multicentric cohorts (14 countries: 89% [26]). This provides strong evidence that the provision of ART to children by nurses at the primary care level is feasible and effective (Table 2), and does not require the presence of paediatric specialists. The steady increase in enrolment of children - annual enrolment more than doubled in two years, from 54 in 2006 to 116 in 2008 - reflects an increase in confidence and skill of nurses to initiate treatment in children, although this remains an important challenge in the programme.

### Innovations to support the expansion and quality of HIV services

Given the extreme poverty and the shortage of health staff in Lesotho, the national HIV/AIDS programme is notable for having introduced a range of innovations that are still absent from the policies of its better resourced neighbours. In 2007, national ART guidelines were revised to raise the threshold of initiation from CD4 counts of less than 200 cells/mm^3 ^to less than 350 cells/mm^3^. The guidelines included a number of treatment innovations, including tenofovir disoproxil fumarate in first-line therapy for adults, a state-of-the-art PMTCT protocol that includes triple therapy for mothers, and early infant diagnosis by DNA polymerase chain reaction testing and initiation of ART for all HIV-positive infants less than 12 months.

Similarly, while many countries in southern Africa have been reluctant to endorse nurse initiation of ART despite the fact that it is recommended by the World Health Organization, Lesotho has permitted nurse initiation since 2006 and it was incorporated formally into the Lesotho National Treatment Guidelines in 2008 [27]. Finally, while the engagement of lay counsellors to support HTC, adherence support and other essential services is still not endorsed by many countries, in Lesotho it has clearly facilitated the expansion of care and contributed to empowering people living with HIV/AIDS.

The early introduction of these innovations is in stark contrast to neighbouring South Africa, where tenofovir is not authorised for routine use in first-line treatment in the public sector. Initiation is delayed until 200 cells/mm^3 ^(for asymptomatic patients), and nurse initiation and lay counsellor administration of HIV rapid tests is stymied: both are provided for in the regulatory framework, but approval has not been forthcoming from the national level.

### Strengthening primary health care to address leading causes of death

There has been considerable debate about the extent to which interventions to address HIV have supported the delivery of primary health care. In such debates, it is important to first consider the burden of disease and how HIV is affecting general health indicators. In Lesotho, HIV/AIDS has contributed to a major increase in mortality - it accounts for at least 60% of all deaths in the country [28] - and decrease in life expectancy and overall negative population growth over the past two decades.

Whereas in previous years, the leading causes of infant and under-five mortality were neonatal and diarrhoeal diseases, 56% of deaths in children are now HIV related [29]. In order to have an impact on childhood mortality in Lesotho, therefore, the single most important interventions are to: treat HIV-positive pregnant women, thereby reducing the risk of vertical transmission; facilitate early diagnosis of HIV in infants; and initiate treatment as soon as possible for infants who are HIV infected.

By integrating comprehensive HIV/AIDS services into the primary health care package, existing health systems in Scott catchment area were better able to address the leading cause of mortality among adults and children (HIV/AIDS itself). In addition, the inputs needed to support HIV services - such as improving nurses' clinical management skills, strengthening laboratory capacity, reinforcing the drug supply system, improving general infrastructure, and strengthening programme management capacity - were, by design, to the benefit of the entire primary health care system in the catchment area.

Clinical management skills of MOHSW nurses in facilities in Scott catchment area were improved through intensive out-of-facility training and in-service clinical mentorship. Training of nurses improved diagnostic and management skills in other common presentations at primary care level (authors' observations). The syndromic management of sexually transmitted infections, diarrhoea and reproductive tract infections, and improved monitoring of children, including assessment of malnutrition, were additional areas, alongside HIV, where the quality of management was improved. A survey done by MSF in 2007 among 47 health centre and hospital nurses trained in HIV/AIDS found that almost all (46) said they felt improved morale and confidence since acquiring skills and tools to provide HIV/AIDS care and treatment

Scott Hospital's laboratory, serving the hospital and health centres, was improved through additional equipment, technical and management training, and additional human resources initially paid for by MSF and now absorbed by Scott Hospital. A mobile specimen collection system enabled clinic patients to forego costly trips to the hospital and allowed nurses and patients to have more timely results. The government, in collaboration with another non-governmental organization, has recently started to implement this system independent of MSF.

Drug supply and management were improved through training and supervision, structural improvements to increase storage capacity, and subsidising additional human resources at the Scott Hospital pharmacy (initially paid for by MSF and now absorbed by Scott Hospital).

Substantial infrastructure improvements have been made to expand capacity for an increased volume of patients, improve patient flow and clinic organization, and address deplorable working conditions for health staff. Priorities were identified through basic audits and discussions with staff and included: providing essential equipment (such as stethoscopes, thermometers and syringes); improving organisational capacity with basic furniture and supplies (such as additional benches, cabinets and patient files and folders); improving conditions (for example, by upgrading radios and providing coal and other materials for heating during winter months); and perhaps most importantly, improving infection control and occupational health practices. The most important addition relating to occupational health was the provision of HIV services for health workers, in light of the fact that death due to HIV has been cited as the number one reason for health care worker attrition in the country [30].

Finally, programme management capacity has been improved. Mobile medical teams have supported improved data recording and reporting, and a simplified cohort monitoring tool was developed using restricted indicators in order to produce quarterly outcomes and empower nurses with feedback about the services they are providing so that they can make necessary improvements to enhance quality of care. Individual clinics are assessed through a quarterly TB/HIV clinic supervision tool, which measures both process and outcome indicators. Supervision visits are now carried out jointly by MSF, Scott Hospital, and district health management teams.

## Conclusion

The MSF-supported programme in Scott catchment area provides further evidence that HIV care and treatment can be provided effectively at the primary care level, to the benefit of primary health care services. It also validates several critical areas for task shifting that are being piloted in many countries in southern Africa and beyond, including nurse-driven ART for adults and children, and lay counsellor-supported testing and counselling, adherence and case management. In addition to rapidly increasing coverage of ART and related services, the programme has managed to incorporate some of the latest national and international guidelines for PMTCT and ART that support important improvements in quality of care.

Given Lesotho's severe resource constraints, the aim of the MSF-supported programme in Scott was to develop a model that was replicable and sustainable in the long term, while meeting ambitious early targets for ART enrolment and ensuring quality of care.

The first phase of the programme, designed for three years, has come to a close and the project has entered a hand-over phase, during which MSF will gradually transfer all responsibilities to the MOHSW and other local health authorities and partners. The real test of whether the objectives of sustainability and replicability of the model have been met will come after the programme has been fully handed over. The ongoing success of a similarly decentralized model of care in rural South Africa that was handed over to the government more than three years ago gives cause for optimism [16].

However, a number of critical clinical and programme-level challenges remain to be addressed. Clinical challenges include: continuing enrolment rates for ART care to keep up with ever increasing needs without compromising quality of care; increasing nurse confidence and skills for paediatric care; increasing the role of lay counsellors to screen stable patients on ART; promoting community-based PMTCT; and continuing to improve diagnosis and management of TB, including smear-negative, extra-pulmonary and DR-TB, as it is the leading cause of death among HIV-positive individuals in the programme and in Lesotho more generally.

At the programme level, key challenges include: ensuring minimum necessary staffing levels, ongoing training and clinical mentorship; assuring an uninterrupted supply of essential HIV medicines, including ARVs; and further boosting programme management capacity.

Partly in recognition of the success of the Scott programme, the Government of Lesotho is reviewing the policies for scaling up task shifting at the national level. Special attention needs to be paid to finding a solution that will guarantee the long-term engagement of the HIV/TB lay counsellors in health service support, as this is a new cadre created to support HIV care. In 2009, efforts were made by numerous international non-governmental organizations working in Lesotho to harmonize their policies for engagement of lay counsellors across the country and to provide recommendations, evidence and input to the MOHSW regarding establishment of a national standard for remuneration, training and core responsibilities of lay counsellors as a first step towards their recognition as a formal health cadre.

Finally, efforts must continue to strengthen the primary care services to accommodate the ever-increasing numbers of patients who need to be initiated on ART in the years to come. Out-of-facility models of care have been found to be effective in other parts of southern Africa [31] and will likely need to be developed to provide support for stable patients with good adherence as a way to make treatment follow up less burdensome for patients and the health system.

## Competing interests

The authors declare that they have no competing interests.

## Authors' contributions

RC, SL and NF wrote the first draft of the manuscript. KH and LK did the statistical analysis. RC, SL, HB, EE, NV, PP, PS, EG, and LM all contributed to the design and implementation of the programme. All authors contributed to subsequent and final drafts of the article.
